# Geochemical Baseline Establishment and Source-Oriented Ecological Risk Assessment of Heavy Metals in Lime Concretion Black Soil from a Typical Agricultural Area

**DOI:** 10.3390/ijerph18136859

**Published:** 2021-06-26

**Authors:** Qi Li, Jinming Zhang, Wen Ge, Peng Sun, Yafen Han, Husen Qiu, Shoubiao Zhou

**Affiliations:** 1 School of Ecology and Environment, Anhui Normal University, Wuhu 241000, China; Liqi@ahszu.edu.cn (Q.L.); zhang_jm2017@163.com (J.Z.); gewen0919@yeah.net (W.G.); 2 School of Environment and Surveying Engineering, Suzhou University, Suzhou 234000, China; sunpeng198608@126.com (P.S.); Hanyafen@ahszu.edu.cn (Y.H.); husenqiu@163.com (H.Q.); 3 National Engineering Research Center of Coal Mine Water Hazard Controlling, Suzhou 234000, China

**Keywords:** reference metal normalization, relative cumulative frequency curve, potential ecological risk, source apportionment, PMF receptor model, Suzhou City

## Abstract

To accurately assess the potential ecological risk posed by heavy metals in lime concretion black soil and quantify the risk contributions from different sources, an investigation of 217 surface soil samples and 56 subsoil samples was performed in the southern part of Suzhou City. Geochemical baseline values of soil heavy metals (Cr, Zn, Pb, Ni, Hg, Cu, Cd, As, Mn and Co) in the study area were calculated as 53.6, 61.5, 19.8, 27.6, 0.08, 18.4, 0.13, 12.9, 416.1 and 11.0 mg/kg, respectively, by using reference metal normalization and cumulative frequency curve methods. Subsequently, four potential sources of soil heavy metals were identified by the positive matrix factorization. Finally, the potential ecological risks arising from the identified sources were determined by the integrated model of positive matrix factorization and Hakanson potential ecological risk index. Results showed that the ecological risk posed by soil heavy metals in the study area ranged from low to moderate level. Hg and Cd were the two largest risk contributors, supplying 36.0% and 30.3% of total risk value. The origin of heavy metals in the soils is mostly related to four sources including agricultural activities, natural dispersion, coal consumption and traffic pollution. Source apportionment of the potential ecological risks revealed that the dominant risk source in the study area was natural dispersion (42.0%), followed by coal related industries (26.5%), agricultural activities (20.4%) and traffic pollution (11.1%). This work gives a clear baseline information of the heavy metal accumulations in lime concretion black soil and provides a successful case study for the source-oriented ecological risk assessment.

## 1. Introduction

Soil is an important component of the natural ecological environment and it is of great value in maintaining plant productivity and supporting human survival [1]. Due to the rapid industrialization and large-scale urbanization, soil pollution has become an increasingly severe problem in recent years. Heavy metal (HM) pollution is widespread in soils and has attached extensive concern because of the high toxicity and non-degradability of HMs, as well as the long-term threat they pose to human lives [2,3]. High content of HMs in soil not only adversely influences soil ecological structure and function, but also causes grave harm to human health through multiple exposure pathways, such as such as food chain transmission, dermal contact and inhalation [4]. Thus, preventing and controlling HM pollution in soil is one of the most urgent problems in today’s society.

To evaluate soil HM pollution, background value (BV) and geochemical baseline value (GBV) are frequently used as references. BV is the HM concentration for a given medium that reflect natural processes uninfluenced by anthropogenic activities [5,6]. It is generally regarded as a reference level for distinguishing between natural element concentrations and anthropologically influenced concentrations [7]. There has not been a well-accepted definition of GBV worldwide till now. Many scholars consider GBV as a measure of given samples in a specific location and time [8,9,10]. Differing from BV, GBV is more inclined to explore the current state of environment and represents the regional present element concentration that consists of natural background and non-point source pollution [11]. Due to the increasing human activities, estimating the “natural background” levels of trace elements in soils has become almost impossible. In this case, GBV can be used as representative of “ambient background” or “actual background” for measuring the present level of environment quality and quantifying the future change of trace elemental concentration in soils. In regard to GBV determination, the methods used in the published reports mainly consist of substitution sample [12], normalization [13,14,15], robust statistical procedures [14,15], and the integration methods combining two or more of the above [16]. The substitution sample method is to use the surface soil samples far away from anthropogenic source or the subsoil samples with minimal disturbance to establish geochemical baseline. The details of normalization method and robust statistical procedures (including relative cumulative frequency, box-whisker plot and iterative methods) are introduced by Zhang et al. [15].

The key issue for solving the problem of soil HM pollution is the identification of contaminants’ provenance. In recent years, receptor models have aroused more and more concern and the applications of them greatly facilitate the quantitative sources apportionment of soil HMs. The common receptor models include chemical mass balance (CMB), positive matrix factorization (PMF), Unmix, etc. [17,18]. Among them, PMF, recommended by the US-EPA, is widely applied, since it does not depend on prior knowledge regarding source profiles [19]. It has been proven to be a useful tool for apportioning HM sources in soils and sediments [20,21]. Recently, more and more attentions are being paid to source-oriented risk assessment related to HMs [22,23,24]. From the perspective of pollution management and control, selecting priority pollution sources should not only depend on the source contributions to HM content, but also consider the toxicities of different HMs. In some cases, the greatest source input to the HM content does not necessarily contributes to the ecological risk [25,26]. Therefore, it should be of great concern to develop the source-oriented ecological risk assessment by combining receptor model with ecological risk index.

Lime concretion black soil (LCBS) is an important Vertisol type in China, and mainly distributed in the plain of northern Anhui Province. This region is one of the most important agricultural regions in China. The environmental quality of LCBS is particularly important because of its close relationship to food security and human health. Therefore, it is necessary to conduct a HM investigation of LCBS to provide baseline and source information on the impact of human activities. Based on the investigation of LCBS in southern part of Suzhou City, this study intends to (a) establish the geochemical baseline of HMs for LCBS in the study area, (b) evaluate the potential ecological risks posed by selected HMs, and (c) quantitatively determine the contributions of various sources to both HM content and ecological risk.

## 2. Materials and Methods

### 2.1. Study Area

The southern part of Suzhou City, including parts of the administrative regions of Yongqiao district, Lingbi and Sixian counties situated at the eastern end of the LCBS area in northern Anhui Province, was selected as the study area. It is between latitude 33°16′ N–33°39′ N and longitude 116°51′ E–118°02′ E, with a total area of 2167 square kilometers. The average annual temperature and precipitation are 14.4 °C and 890 mm. The study area is covered by LCBS (Vertisol) and Yellow-tide soil (Fluvisol). LCBS is dominant soil type in the study area, accounting for 84.6% of the total land area. It is characterized with low organic C concentrations (<0.87%), high montmorillonite contents (>12%) and poor water-air permeability [27]. Besides, remarkable acidification tendency of LCBS has been found in the study area, which may enhance activities of toxic elements and increase potential risk of HM pollution in the soil [28]. Suzhou City has developed traditional agriculture and is the important region of grain and fruit production in Anhui Province. The main grain crops in the study area are wheat and maize. In addition, Suzhou City is abundant in coal resources and five coal mines with annual coal output of 15 million tons are concentrated in the western of the study area. In the mining area, there are many coal-related industries, such as gangue-fired power plants, brick fields, cement factories and transportation enterprises.

### 2.2. Sample Collection and Analytical Method

**Figure 1:**
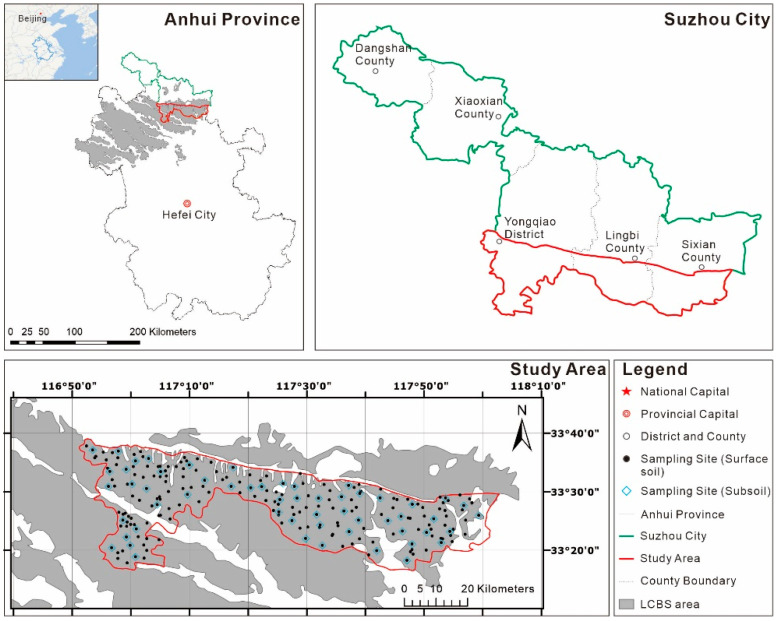
Study area and sampling sites.

A total of 217 surface soil (0–20 cm) samples and 56 subsoil (20–40 cm) samples were collected from the study area in August 2020. The surface soil sampling was pre-designed at a density of about one site every 10 square kilometers. In comprehensive consideration of soil type and land utilization, locations of 217 sites were finally determined based on the principles of randomness and homogeneity to represent the entire study area (Figure 1). Furthermore, fifty-six of these sampling sites were randomly chosen for collection of the subsoil samples. Coordinates of all the sampling sites were recorded by a hand-held Global Positioning System (GPS) device, and the spatial distribution of the sampling sites is shown in Figure 1. In each surface soil or subsoil sampling site, three sub-samples (about 150–200 g in weight per sub-sample) were collected using the diagonal multi-point sampling method (scale 100 × 100 m) and then mixed evenly to acquire a homogenized sample of about 500 g in weight. All the soil samples were stored in plastic self-sealing bags and then transported to the laboratory.

After being air-dried at room temperature (25 °C), the soil samples were ground with agate mortar that could pass through a 0.149 mm nylon sieve. The samples were digested with HClO_4_-HCl-HF for analysis of As and Hg, and with HClO_4_-HNO_3_-HF for analysis other elements. As and Hg were determined using a hydride generation atomic fluorescence spectrometry (HG-AFS, Model PF5, Purkinje General Instrument, Beijing, China), the other elements were measured using an inductively coupled plasma optical emission spectrometry (ICP-OES, Model PF5, Purkinje General Instrument). The accuracy of the elements analysis was controlled using soil standard reference material GSS-16 (GBW07430). One control sample was set in each batch of digestion for every 30 samples. The recovery of each element was found in the range of 100 ± 15% (Table 1), indicating the measurement accuracy in this study was satisfactory.

### 2.3. Calculation Methods of GBV

Geochemical baseline values (GBVs) of ten HMs (Cr, Zn, Pb, Ni, Hg, Cu, Cd, As, Mn, Co) were determined using subsoil samples and two calculation methods, including reference metal normalization and relative cumulative frequency curve. The reference metal normalization method can be expressed as an equation established by the correlation between the studied metals and reference elements [29]:

(1) $$C_{m}=a{\bar{C}}_{N}+b$$

where *C_m_* represents the GBV of the studied HM (mg/kg), *C_N_* is the average concentration of the reference element, *a* and *b* are the regression coefficient and constant, respectively. Concretely speaking, linear regression is firstly conducted between the studied HM and the reference element. Based on fitting relation, samples falling outside the scope of 95% confidence intervals are eliminated until no outliers remained. Then, the regression parameters *a* and *b* are determined. Finally, GBVs of the studied HMs are calculated by substituting average concentrations of the reference elements into the equation.

For the relative cumulative frequency curve, the X-axis is the HM concentrations and the Y-axis represents their corresponding cumulative frequency. After the curve is established, inflexion point can be determined under linear regression model with the criterion of *p* < 0.05 and R^2^ > 0.95 [15]. Generally, one or two inflexion points may be found on the curve. If there is only one inflexion point, the GBV was calculated as the average value of all data below the inflexion point. In the case of two inflexion points, the curve shape between two bends is considered a critical factor. If the middle part of the cumulative distribution curve is similar to the forepart (or the following part), data involved in calculation should be included the part before the lower inflexion point (or before the upper inflexion point).

### 2.4. PMF Receptor Model

PMF, a multivariate receptor model for source apportionment, was originally proposed by Paatero [30]. It decomposes raw data matrix into three components including factor contribution matrix, factor profile matrix and residual error matrix using the multilinear engine-2 tool (ME-2):

(2) $$x_{ij}=\overset{p}{\underset{k=1}{\sum }}g_{ik}f_{kj}+e_{ij}$$

where, *x_ij_* is the raw concentration of *j* th element in sample *i*; *g_ik_*, *f_kj_* and *e_ij_* represent the factor contribution matrix, the factor profile matrix and the residual error matrix, respectively. *p* is the source quantity. This equation can be solved through minimizing the target function Q:

(3) $$Q=\overset{m}{\underset{i=1}{\sum }}\overset{n}{\underset{j=1}{\sum }}{\left(\frac{e_{ij}}{u_{ij}}\right)}^{2}$$

where *u_ij_* is uncertainty in the *j* th element for sample *i*, and it can be calculated based on the method detection limit (*MDL*) and error fraction measured by standard reference materials [31]:

(4) $$\mathrm{If}\;c\leq MDL,\;u_{ij}=\frac{5}{6}\times MDL$$

(5) $$\mathrm{If}\;c>MDL,\;u_{ij}=\sqrt{{\left(Errorfraction\times c\right)}^{2}+MDL^{2}}$$

### 2.5. Potential Ecological Risk Assessment

Potential ecological risk caused by HMs in the study area was assessed using Hakanson potential ecological risk index (HPERI). This index (*RI*) was computed by summing individual potential risk factor (*E_j_*) using Equations (6) and (7):

(6) $$RI=\overset{n}{\underset{j=1}{\sum }}E_{j}$$

(7) $$E_{j}=T_{j}\times \frac{C_{j}}{C_{j\text{-}GBV}}$$

here, *T_j_* is toxic response factor of the *j* th metal (defined as Zn = Mn = 1, Cr = 2, Pb = Ni = Cu = Co = 5, As = 10, Cd = 30, Hg = 40), *C_j_* is concentration of the *j* th metal in soils and *C_j-GBV_* represents GBV of the *j* th metal in the study area.

For quantitatively risk grading, the classification standards for *E_j_* are: *E_j_* < 40 (low risk), 40 ≤ *E_j_* < 80 (moderate risk), 80 ≤ *E_j_* < 160 (considerable risk), 160 ≤ *E_j_* < 320 (high risk) and ≥ 320 (very high risk), while for *RI* are: *RI* < 150 (low risk level), 150 ≤ *E_j_* < 300 (moderate risk level), 300 ≤ *E_j_* < 600 (severe risk level) and ≥600 (serious risk level) [32].

Furthermore, the PMF-HPERI model combining PMF model with HPERI was used for source apportionment of the potential ecological risk. It can quantitatively determine the risk values and contributions of various sources by predicted source profiles derived from PMF. The used equations are as follows:

(8) $$E_{j}^{k}=\frac{1}{n}\overset{n}{\underset{i=1}{\sum }}(T_{j}\times \frac{C_{ij}^{k}}{C_{j\text{-}GBV}})=\frac{1}{n}\overset{n}{\underset{i=1}{\sum }}(T_{j}\times \frac{F_{ij}^{k}\times C_{ij}}{C_{j\text{-}GBV}})$$

(9) $$R^{k}=\frac{\sum_{j=1}^{n}E_{j}^{k}}{RI}\times 100\%$$

where, *E_j_^k^* is potential risk value of the *j* th metal released from source *k*, *n* is the soil sample quantity, *C_ij_^k^* and *F_ij_^k^* are the calculated concentration and its corresponding contribution of the *j* th metal released from source *k* in sample *i*, respectively, *C_ij_* is the predicted concentration of the *j* th metal in sample *i*, *R^k^* is the risk contribution rate of the source *k* to *RI*.

### 2.6. Data Statistical Analysis

Descriptive statistics, such as mean, median, standard deviation, coefficient of variation, minimum and maximum, were applied in the characterization of HM concentrations in soils. Data analyses were performed using the Excel 2017 (Microsoft Company, Redmond City, WA, USA) and SPSS 13.0 (International Business Machines Corporation, Armonk City, NY, USA) software packages. The PMF 5.0 model recommended by US-EPA was used to perform source apportionments of HMs in soils. Maps associated with spatial distribution were achieved using the method of ordinary kriging in ArcGIS 10.2 (Environmental Systems Research Institute, Inc., Redlands City, CA, USA).

## 3. Results and Discussion

### 3.1. General Descriptions of HM Concentrations in the Surface Soils

The statistical characteristics of ten HMs in the surface LCBSs collected from the study area are shown in Table 2. From Table 2, it can be seen that the mean concentrations of Zn, Ni, Hg, Cu, Cd and As were 83.1, 35.1, 0.10, 24.3, 0.17 and 17.1 mg/kg, higher than the soil background values of Anhui Province. Especially, the maximum of Hg, Cd and As concentrations were 20.6, 5.8 and 3.5 times of their respective background value, indicating the anthropologic accumulation in outlier. The average concentrations of Cr, Pb, Mn and Co were slight lower than the soil background values.

The coefficient of variation (CV) was applied to show the spatial variation degree of soil element’s concentrations in the study area. It can be graded according to its value as: CV ≤ 10% with low spatial variation, 10% < CV ≤ 30% with moderate spatial variation, and CV > 30% with high spatial variation. Among the investigated elements, Hg had the highest CV value of 38.6%, followed by Cd (31.4%), and they were categorized as high spatial variation class. All the other elements were categorized as moderate spatial variation class, and their CV values were followed the descending order of Zn (29.9%) > Cu (29.7%) > Ni (28.9%) > As (25.1%) > Pb (23.0%) > Cr (18.7%) > Mn (17.2%) > Co (12.8%).

### 3.2. GBVs Determination

**Figure 2:**
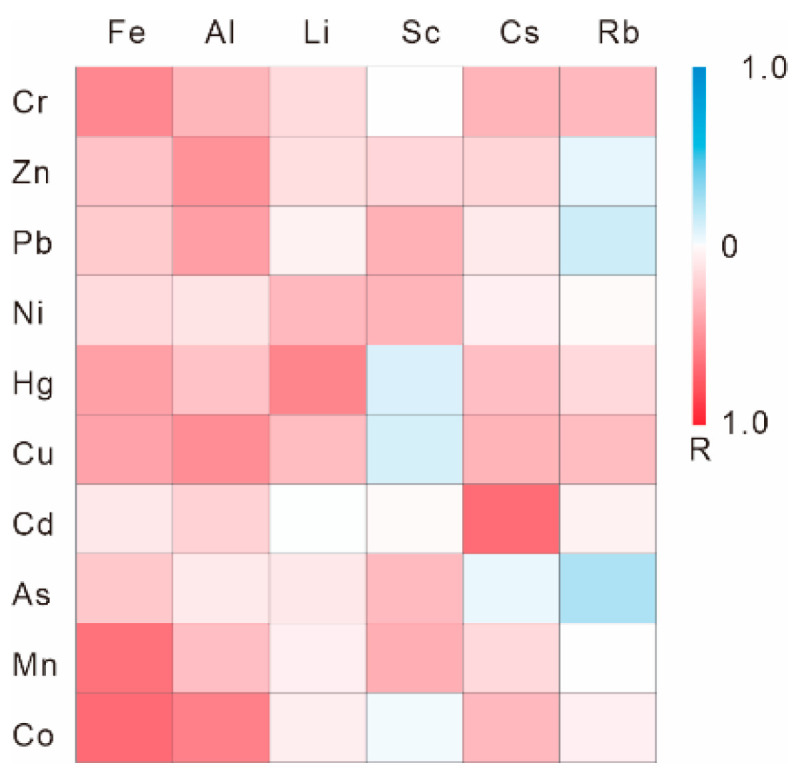
Pearson correlation analysis between the six reference elements and the studied HMs.

Selection of the referenced element is an essential prerequisite for performing the reference metal normalization method. Many reference elements, such as Al, Fe, Ti, Rb, Li and Sc, have been proposed as the suitable normalizers for establishing geochemical baseline, due to their relatively weak chemical activity and migratory ability. In this study, six reference elements (Al, Fe, Li, Sc, Rb and Cs) were considered as the candidates of the normalizer, and the Pearson correlation coefficients between these candidates and the studied HMs were compared (Figure 2). The results indicated that Fe had the most excellent correlation with Cr, Mn and Co, with the correlation coefficient values (R) being 0.56, 0.65 and 0.68; Al showed most closely relationship with Zn, Pb and Cu, with the R values being 0.51, 0.45 and 0.53, respectively; Sc had the highest R values with Ni (0.35) and As (0.32); Li was well correlated with Hg (R = 0.57), whereas Cs displayed the highest correlation coefficient value with Cd (R = 0.67).

**Figure 3:**
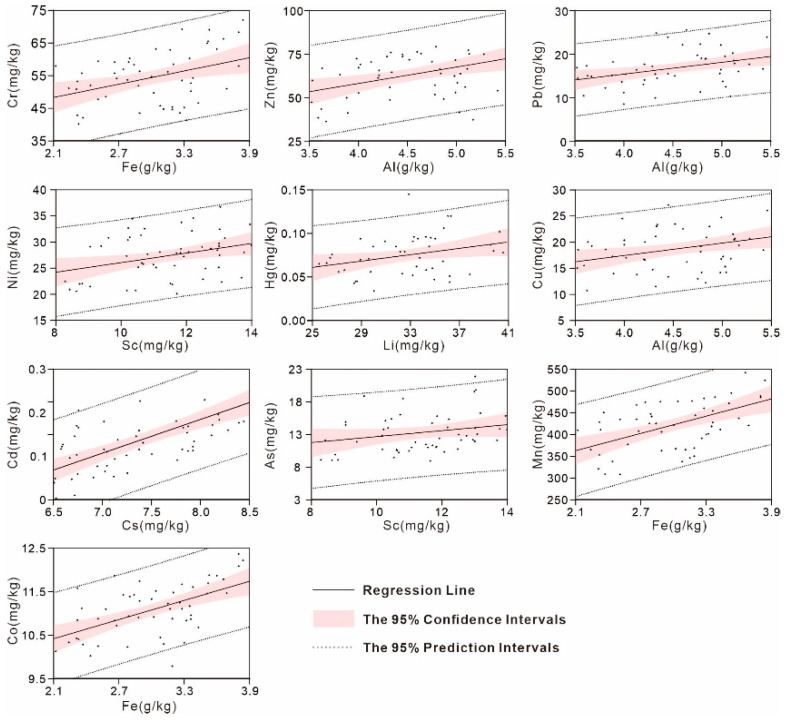
Linear regression analyses between the studied HMs and their optimal normalizers.

Reference elements with the highest correlation coefficients were identified as the optimal normalizers for their corresponding HMs, and linear regression analyses were also performed after eliminating the outlier samples by the method of double standard deviation (Figure 3). Generally, the data points falling inside the 95% confidence interval of the regression line can be regarded as natural sediments without any anthropogenic disturbance, therefore representing the basis for predicting the GBVs [33]. According to the procedure mentioned in Section 2.3, the regression equation between each HM and its normalizer was established by iteratively eliminating the samples lying outside 95% confidence intervals, as shown in Table 3. Then, the GBVs obtained by reference metal normalization method were 53.7, 63.9, 20.3, 27.8, 0.08, 18.5, 0.15, 13.5, 414.8 and 10.9 mg/kg for Cr, Zn, Pb, Ni, Hg, Cu, Cd, As, Mn and Co, respectively.

**Figure 4:**
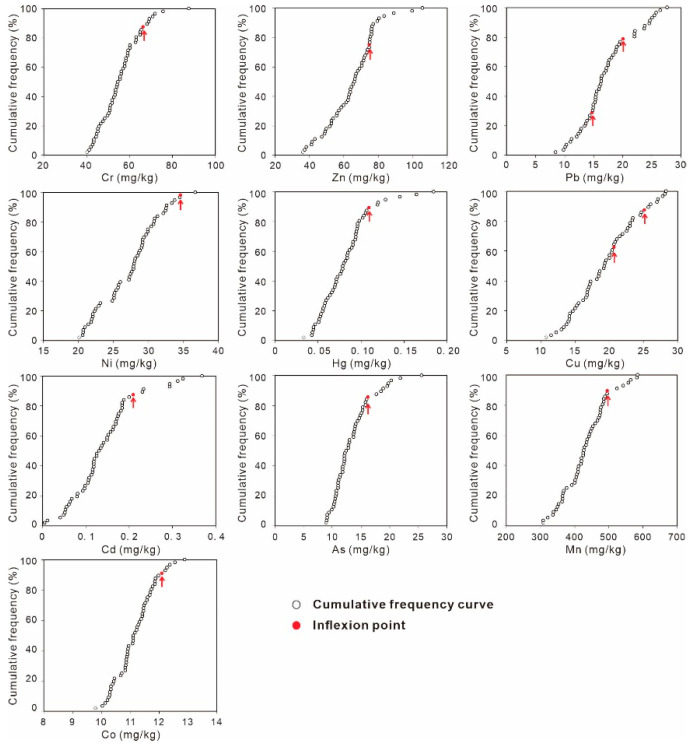
Relative cumulative frequency curves of HMs in the soils of study area.

The cumulative frequency curves for the selected HMs show that there was a single inflexion point on the curves for Cr, Zn, Ni, Hg, Cd, As, Mn and Co, but two inflexion points could be recognized in the curves of Pb and Cu (Figure 4). For both Pb and Cu, the curve shape between two the inflexion points was similar to that after the upper outliers. Accordingly, all the data before the upper inflexion point were involved in the calculation of GBVs. The GBVs obtained by cumulative frequency curve method were 53.5, 59.1, 19.2, 27.3, 0.08, 18.3, 0.12, 12.6, 417.4 and 11.1 mg/kg for Cr, Zn, Pb, Ni, Hg, Cu, Cd, As, Mn and Co, respectively.

As shown in Table 3, the final GBVs in the study area, calculated as the average values of the results obtained from the above two methods, were 53.6, 61.5, 19.8, 27.6, 0.08, 18.4, 0.13, 12.9, 416.1, 11.0 mg/kg for Cr, Zn, Pb, Ni, Hg, Cu, Cd, As, Mn and Co, respectively. Compared with the BVs of Anhui Province (Table 2), the GBVs for Hg, Cd and As in this study are much higher, whereas for other elements the GBVs are obviously lower. This result suggests that pollution assessment using LBVs as benchmark may misestimate the contamination levels of HMs in LCBS. Therefore, the obtained GBVs were used for the subsequent potential ecological risk assessment of surface soil HMs in this study.

### 3.3. Source Analysis by PMF

**Figure 5:**
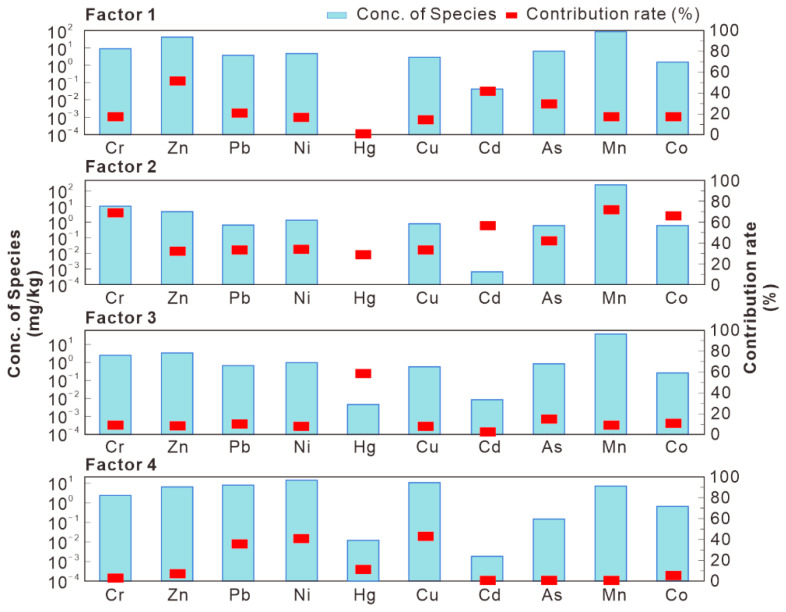
Concentration and contribution rate of HMs from various PMF-source factors.

**Figure 6:**
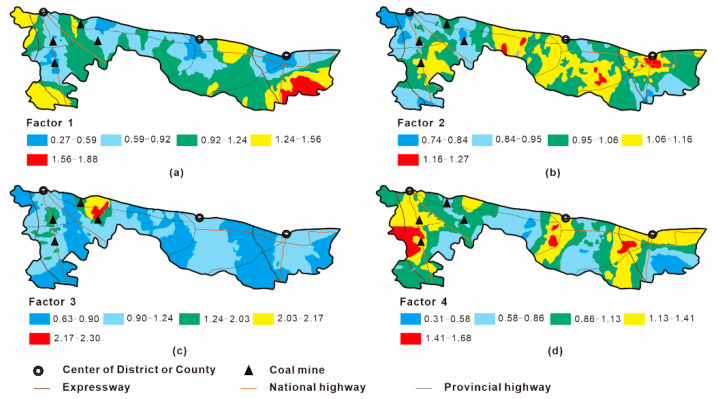
Spatial distributions of the four PMF-source factors in the investigated area. (**a**) Factor 1: agricultural activities; (**b**) Factor 2: natural source; (**c**) Factor 3: coal related industries; (**d**) Factor 4: traffic pollution.

The concentration data of some HMs in 217 samples and uncertainty data associated with these concentrations were imported into the PMF 5.0 model, and then 50 iterative operations were performed. Finally, the best solution with the lowest Q value (6887.8) was obtained, including four factors. The R^2^ values of all the studied HMs were greater than 0.7 and the ratios of P/O (Predicted/Observed) were closed to 1.0, suggesting a reliable fitting result (Table 4). The contributions of various factors to the accumulation of ten HMs in soils are illustrated in Figure 5; moreover, the spatial distributions of four factors in the investigated area are drawn using the ordinary kriging method and presented in Figure 6.

Factor 1 prevails for Zn, Cd and As, with contribution rates of 51.0%, 40.4% and 41.8%, respectively, and it may represent the contamination sourced from agricultural production. LCBS is the predominant agricultural soil type in northern Anhui Province and it is characterized by infertility and shortage of organic matter [27]. In the study area, application of chemical fertilizers and pesticides are the most widespread measures to ameliorate soil fertility and improve grain yield. The agricultural statistical data indicated that the annual application amounts of chemical fertilizers and pesticides in Suzhou City were approximately 300 and 20 kg/hm^2^, respectively, whereas most of them were unable to be fully utilized and finally lost into the surroundings.

Zn is abundant in chemical fertilizers, since it can promote growth and enhance disease resistance for plants. Previous studies also found that phosphate fertilizer is an important carrier for cadmium and arsenic [34,35]. The Cd concentrations in agricultural soils were significantly correlated with the amount of fertilizer application [36]. Inorganic arsenic-containing pesticides are a type of effective bactericide and frequently used in prevention and treatment of plant diseases [37]. Therefore, it is inevitably that Zn, Cd and As would sneak into soils in the course of fertilization and pesticide spraying. Furthermore, the spatial distribution of factor 1, as shown in Figure 6a, revealed that the dark areas with high values were mainly located in the suburbs, which was consistent with the distributions of farmlands and vegetable field in the investigated area. Consequently, we concluded that factor 1 represents the impact of agricultural activities.

Factor 2 is relevant for Cr (69.5%), Mn (72.1%) and Co (66.2%). Contribution rates of the other metals were in the range of 29.5%–59.2%. Generally, the grouping of Cr, Mn and Co points to a likely natural origin, which has been verified by the investigations conducted in the Drava Valley and the Piemonte region [38,39]. In this study, Cr, Mn and Co in the surface soils show mean concentrations lower than values characterizing their respective background (Table 2), indicating that these metals are lacking obvious artificial sources, mostly controlled by parent materials. This result is consistent with that of the previous small-scale investigation conducted in a densely populated campus form the present study area [40]. Hence, the factor 2 could be considered as a natural source.

Factor 3 is basically critical for Hg, with contribution rates of 58.7%. Hg had the highest CV value of 38.6% (Table 2), revealing that its dispersion is greatly influenced by human activities. From Figure 6c, it can be seen that hotspots with high values of factor 3 were mainly located around the coal mines. It is well-established that smelting, fossil fuel mining and combustion are the most important anthropogenic sources of Hg in China. Fei et al. reported that more than 90% of Hg in surface soil samples collected from Hangzhou City was discharged from coal mining and smelting industries [41]. In the western of the study area, five coal mines had operated for over twenty years, and over 15 million tons of coal and 0.75 million tons of coal gangue were produced annually. Six gangue-fired power plants and dozens of small coal-fired factories were also located around these mines, consuming large amounts of coals and coal gangues annually. During these processes, the Hg existed in coal or its by-product could be released readily into atmosphere, and then entered into soils through atmospheric dry/wet deposition [42]. This could be corroborated by the previous research which found severe Hg pollution in atmospheric dust in this coal mining area, with the average concentration of 1.29 mg/kg [43]. Therefore, factor 3 was deemed to be the pollution source associated with consumption of coal and coal gangue.

Factor 4 has higher contributions to Pb (36.8%), Ni (41.7%) and Cu (43.4%). These metals are commonly regarded as fingerprints of traffic pollution. It has been confirmed that substantial amounts of Pb, Ni and Cu in the soils flanking traffic lines came from automobile combustion or the dust produced by automobile tire wear [44,45]. The present study area has convenient and mature traffic network, including two expressways, four national highways and four provincial highways. Figure 6d revealed that the high-value regions of factor 4 were inclined to be distributed in the sides of the traffic lines. The values of factor 4 in the urban districts and their nearby areas were also obviously higher than those in the suburbs. These trends indicate that factor 4 was very likely well correlated with the traffic volume. Therefore, factor 4 could be considered to represent traffic pollution source.

### 3.4. Potential Ecological Risk Assessment

According to the Equations (6) and (7), individual potential ecological risk factor (E_j_) and comprehensive potential ecological risk index (RI) of HMs in the soils sampled for the study area were calculated and listed in Table 5. The mean values of E_j_ for each HM decreased in an order of Hg > Cd > As > Pb > Cu > Ni > Co > Cr > Zn > Mn. Based on the classification criteria of Hakanson, the mean E_j_ value of Hg (45.7) belonged to the moderate single potential ecological risk, whereas the mean E_j_ values of the other nine metals were less than 40, suggesting that these metals in the studied area were at the level of low ecological risk. Nevertheless, the maximum E_j_ value of Cd had reached 125.6, indicating that some soil samples bear moderate to strong potential ecological risks posed by Cd pollution.

The comprehensive potential ecological risk index (RI) reflects the general situation of pollution caused by the simultaneous presence of the ten HMs. As shown in Table 5, the mean RI value was 127.0, belonging to the class of low ecological risk. Regarding its composition, Hg is the greatest contributor, followed by Cd. These two elements supplied 36.0% and 30.3% of the total ecological risk values, and the contribution rates of the other metals were all below 11.0%. The RI values of all the 217 soil samples ranged from 89.3 to 283.7, showing low to moderate ecological risk in the study area.

**Figure 7:**
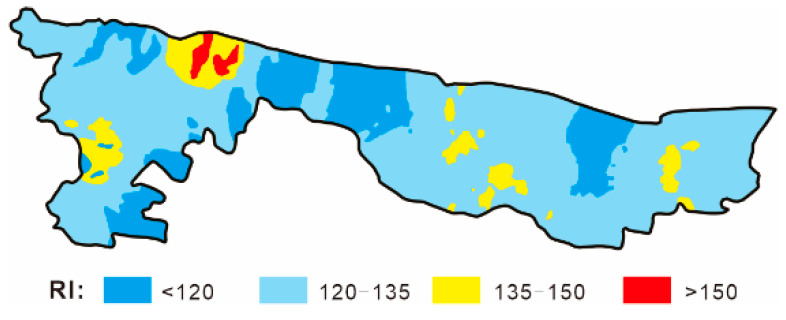
Spatial distribution of RI value in the investigated area.

In order to clarify the distribution of RI value in the investigated area, a spatial interpolation was conducted by the ordinary kriging tool in Acgis 10.2, and the result was illustrated in Figure 7. From Figure 7, soils with moderate ecological risk (RI > 150) were mainly distributed in the two coal mining area located at the southeast of Yongqiao District, where coal exploitation activities lasted over fifty years, whereas soils in the remaining areas carried a low ecological risk. This is consistent with results from a recent research on the overlapped areas of farmland and coal resources in northern Xuzhou City (about 70 km far away from our study area), where the low to moderate ecological risk posed by HMs were also found in soils [46]. Thus, it could be concluded that the soil in the coal mining area had been polluted locally, and the targeted ecological restoration should be proposed to be conducted. Besides, it was noteworthy that there was also a considerable area of land (nearly 110 km^2^) with the RI values ranging from 135 to 150, equivalent to 90% to 100% of the critical value (150). These regions would be very likely to slide into the class of moderate ecological risk with the further accumulation of HMs in soil. Appropriate engineering and ecological measures are necessary to be taken to control the concentrations of soil HMs in the areas.

### 3.5. Source Apportionment of Ecological Risk

Finding out the source apportionment of ecological risk can provide the direct basis for select priority pollution sources from perspective of risk management and control. In this study, PMF-HPERI model was used to calculate the values and proportions of ecological risk contributed from various sources (Table 6). It can be seen from Table 6 that the risk values (E^k^_j_) from different sources varied. Factor 2 (natural source) accounted for the highest proportion (42.0%) of the total ecological risk, followed by 26.5% from factor 3 (source of coal consumption) and 20.4% from factor 1 (agricultural source), whereas factor 4 (traffic pollution source) accounted for only 11.1% of the total ecological risk. Therefore, natural source was the most important factor contributing to ecological risk associated with soil HMs. Moreover, the sum of risk contribution rates from factor 1, factor 3 and factor 4 came up to 58.0%, showing that anthropogenic sources, especially the activities associated with coal consumption and agricultural production, were also the main risk contributors.

In the past decade, receptor models, such as CMB, PMF and Unmix, have been verified to be useful tools for quantitatively studying source apportionment of trace metal’s concentration in soils [25]. Recently, the relationship between source and ecological or human health risk is attracting more and more interests from academia, as considering only the source contribution to the trace metal content while ignoring the difference between toxicities of different metals would not be sufficient for regional environmental management. Several studies focusing on the source apportionment of human health risk arising from HMs has been published [22,23,24,26]. However, as far as we know, the research combining receptor model with potential ecological risk assessment for risk source apportionment of soil trace metals is seldom reported. In view of this, the present work is a good complement to the current research deficiency, and provides a successful case study for the source-oriented ecological risk assessment.

## 4. Conclusions

Descriptive analyses indicated that the mean concentrations of Zn, Ni, Hg, Cu, Cd and As in the collected surface soils were higher than the soil background values of Anhui Province, and furthermore Hg and Cd showed stronger spatial variability. Using reference metal normalization and cumulative frequency curve methods, the GBVs of HMs in LCBS of the study area were determined as 53.6, 61.5, 19.8, 27.6, 0.08, 18.4, 0.13, 12.9, 416.1 and 11.0 mg/kg for Cr, Zn, Pb, Ni, Hg, Cu, Cd, As, Mn and Co, respectively.

PMF model was successful in quantitatively resolving four independent sources of soil HMs in the study area, including natural pedogenesis, consumption of coal and coal gangue, agricultural activities and traffic pollution. Natural source contributed 69.5%, 72.1% and 66.1% of Cr, Mn and Co concentrations, respectively. Coal consumption dominated Hg content with the contribution of 58.7%. Agricultural activities controlled 51.0% of Zn, 40.4% of Cd, and 41.8% of As, while traffic pollution was associated with 36.8% of Pb, 41.7% of Ni and 43.4% of Cu.

According to the result of potential ecological risk assessment, the RI values in the study area ranged from 89.3 to 283.7, indicating low to moderate ecological risk. Soils with moderate ecological risk mainly distributed in two coal mining area, whereas the other areas carried a low ecological risk. Among the measured HMs, Hg and Cd were the main risk contributor, supplying 36.0% and 30.3% of the mean RI value in soils. Based on the integrated PMF-HPERI model, source apportionment of the ecological risk posed by HMs was revealed quantitatively. The contribution rates of the four sources followed the decreasing order of natural source (42.0%), coal consumption (26.5%), agricultural source (20.4%) and traffic source (11.1%).

## Figures and Tables

**Table 1:** Measured values and recovery of standard reference materials GSS-16 (GBW07430).

Items		Cr	Zn	Pb	Ni	Hg	Cu	Cd	As	Mn	Co
Reference value (mg/kg)		67	100	61	27.4	0.46	32	0.25	18	441	13.6
Measured values (mg/kg)	nN	10	10	10	10	10	10	10	10	10	10
	Max	71.7	108	66.5	28.9	0.48	33.0	0.28	18.9	481	14.0
	Min	62.1	91	58.2	25.0	0.41	30.0	0.23	16.2	415	12.9
	Mean	67.6	99	62.3	27.2	0.45	31.1	0.26	17.7	454	13.5
Recovery (%)	Max	107	108	109	105	105	103	113	105	109	103
	Min	93	91	95	91	89	94	93	90	94	95
	Mean	101	99	102	99	98	97	105	98	103	99

**Table 2:** Statistics characteristics of HM concentrations in the surface soils (n = 217).

Element	Mean (mg/kg)	Median (mg/kg)	SD ^1^ (mg/kg)	CV ^2^ (%)	Minimum (mg/kg)	Maximum (mg/kg)	BV ^3^ (mg/kg)
Cr	60.3	60.0	11.3	18.7	38.5	93.5	66.5
Zn	83.1	79.9	24.8	29.9	43.0	127.1	62.0
Pb	22.4	21.2	5.2	23.0	14.9	46.2	26.6
Ni	35.1	31.6	10.1	28.9	18.2	62.8	29.8
Hg	0.10	0.09	0.04	38.6	0.04	0.46	0.033
Cu	24.3	23.3	7.2	29.7	11.2	52.4	20.4
Cd	0.17	0.16	0.05	31.4	0.06	0.56	0.097
As	17.1	16.6	4.3	25.1	9.7	31.1	9.0
Mn	437.0	430.0	75.2	17.2	279.8	621.7	530
Co	10.7	10.6	1.4	12.8	7.3	15.0	16.3

^1^ Standard deviation; ^2^ Coefficient of variation; ^3^ Soil background values of Anhui Province.

**Table 3:** The regression equations and GBVs for the studied HMs.

Element	Reference Metal Normalization Method				Cumulative Frequency Curve (mg/kg)	Final Value (mg/kg)
	Regression Equation	R^2^	*p*	Baseline Values (mg/kg)		
Cr	Cr = 3.45 × Fe + 43.80	0.82	0.01	53.7	53.5	53.6
Zn	Zn = 9.36 × Al + 21.44	0.83	0.01	63.9	59.1	61.5
Pb	Pb = 2.29 × Al + 6.31	0.88	0.01	20.3	19.2	19.8
Ni	Ni = 0.98 × Sc + 16.07	0.83	0.01	27.8	27.3	27.6
Hg	Hg = 0.002 × Li + 0.025	0.77	0.01	0.08	0.08	0.08
Cu	Cu = 2.40 × Al + 7.90	0.83	0.01	18.5	18.3	18.4
Cd	Cd = 0.06 × Cs − 0.30	0.96	0.01	0.15	0.12	0.13
As	As = 0.37 × Sc + 9.01	0.67	0.01	13.5	12.6	12.9
Mn	Mn = 33.73 × Fe + 316.79	0.75	0.01	414.8	417.4	416.1
Co	Co = 0.69 × Fe + 6.96	0.93	0.01	10.9	11.1	11.0

**Table 4:** The R^2^ and P/O values obtained from PMF model in this study.

Items	Cr	Zn	Pb	Ni	Hg	Cu	Cd	As	Mn	Co
R^2^	0.98	0.90	0.80	0.88	0.99	0.89	0.75	0.95	0.99	0.97
P/O	0.87	0.94	0.86	0.83	0.99	0.85	0.66	0.94	0.99	1.22

**Table 5:** Results of potential ecological risk assessment HMs in the soil samples.

Items	E_j_ ^1^										RI ^2^
	Cr	Zn	Pb	Ni	Hg	Cu	Cd	As	Mn	Co	
Mean	2.2	1.4	7.1	6.4	45.7	6.6	38.5	13.3	1.1	4.9	127.0
SD	0.4	0.4	1.6	1.8	16.8	2.0	12.1	3.3	0.2	0.6	21.4
Minimum	1.4	0.7	4.7	3.3	16.7	3.0	12.6	7.5	0.7	3.3	89.3
Maximum	3.5	2.1	14.6	11.4	218.6	14.2	125.6	24.1	1.5	6.8	283.7
Percentage (%)	1.8	1.1	5.6	5.0	36.0	5.2	30.3	10.5	0.8	3.8	

^1^ Individual potential risk factor; ^2^ Potential ecological risk index.

**Table 6:** The potential ecological risk from different sources.

Element	E_j_^k^			
	Factor 1	Factor 2	Factor 3	Factor 4
Cr	0.4	1.6	0.2	0.1
Zn	0.7	0.4	0.1	0.1
Pb	1.3	2.4	0.7	2.6
Ni	1.0	2.1	0.5	2.6
Hg	0.1	14.1	28.2	5.5
Cu	1.0	2.2	0.5	2.8
Cd	15.1	21.3	0.9	0.0
As	5.5	5.6	2.0	0.1
Mn	0.2	0.8	0.1	0.0
Co	0.8	3.2	0.5	0.3
Total	26.1	53.6	33.9	14.2
R^k^ (%)	20.4	42.0	26.5	11.1

## Data Availability

The data presented in this study are available on request from the corresponding author.
